# Combined usage of serodiagnosis and O antigen typing to isolate Shiga toxin-producing *Escherichia coli* O76:H7 from a hemolytic uremic syndrome case and genomic insights from the isolate

**DOI:** 10.1128/spectrum.02355-23

**Published:** 2023-12-04

**Authors:** Kenichi Lee, Atsushi Iguchi, Chikako Terano, Hiroshi Hataya, Junko Isobe, Kazuko Seto, Nozomi Ishijima, Yukihiro Akeda, Makoto Ohnishi, Sunao Iyoda, EHEC Working Group in Japan, Yo Morimoto

**Affiliations:** 1 Department of Bacteriology I, National Institute of Infectious Diseases, Tokyo, Japan; 2 Department of Animal and Grassland Sciences, Faculty of Agriculture, University of Miyazaki, Miyazaki, Japan; 3 Department of Nephrology and Rheumatology, Tokyo Metropolitan Children’s Medical Center, Tokyo, Japan; 4 Department of Nephrology, Aichi Children’s Health and Medical Center, Aichi, Japan; 5 Department of Bacteriology, Toyama Institute of Health, Imizu, Toyama, Japan; Taichung Veterans General Hospital, Taichung, Taiwan

**Keywords:** Shiga toxin-producing *Escherichia coli*, immunomagnetic separation, immunoserology, hemolytic uremic syndrome, genome analysis

## Abstract

Minor O-serogroups of Shiga toxin-producing *Escherichia coli* (STEC) can cause severe clinical complications in humans, including hemolytic uremic syndrome (HUS). However, detection and isolation of these minor serogroups of STEC are challenging due to the lack of specific isolation methods. Here, we present a case of HUS in which STEC was not isolated by routine diagnostic tests for the major serotypes. Therefore, we tried a new diagnostic and isolation method that combines PCR screening, immunomagnetic bead separation, and serum agglutination tests and successfully isolated STEC O76. Subsequent genomic analyses of the STEC O76 isolates revealed that several isolates of this serogroup carrying *stx2* were related to severe infections. The complete genome of the HUS-derived isolates provided two important implications. First, using a complete genome as a reference in core genome single nucleotide polymorphism analysis leads to the highest resolution of the analysis. Second, the HUS-derived STEC O76:H7 possessed two copies of Stx2a prophages, and one of them showed a “prophage integrating into prophage” structure, as described in STEC O145:H28. These results demonstrate that our detection methods contribute to the diagnosis and isolation of minor serogroups of STECs and complete genomic analyses can illuminate the pathogenic potential of STECs.

**IMPORTANCE:**

Hemolytic uremic syndrome (HUS) is a life-threatening disease caused by Shiga toxin-producing *Escherichia coli* (STEC) infection. The treatment approaches for STEC-mediated typical HUS and atypical HUS differ, underscoring the importance of rapid and accurate diagnosis. However, specific detection methods for STECs other than major serogroups, such as O157, O26, and O111, are limited. This study focuses on the utility of PCR-based O-serotyping, serum agglutination tests utilizing antibodies against the identified Og type, and isolation techniques employing antibody-conjugated immunomagnetic beads for STEC isolation. By employing these methods, we successfully isolated a STEC strain of a minor serotype, O76:H7, from a HUS patient.

## INTRODUCTION

Shiga toxin-producing *Escherichia coli* (STEC) is an important foodborne pathogen worldwide (1). The primary reservoirs of STEC are ruminants, such as cattle, sheep, and goats (2, 3). Contaminated beef, raw milk, and fresh produce are often identified as routes of transmission to humans. More than 3,000 cases of infection, including asymptomatic carriage, are reported annually in Japan (4). The major serogroups of STEC isolates in Japan are O157, O26, O103, O111, O121, O145, and O91 according to the number of isolates (4). The clonality of these isolates is monitored using multilocus variable-number tandem-repeat analysis and whole-genome sequencing to detect links among isolates and nationwide outbreaks (5 – 9).

In humans, STEC causes a range of symptoms, including diarrhea, hemorrhagic colitis, and life-threatening hemolytic uremic syndrome (HUS). The majority of HUS cases are caused by serogroup O157, followed by O26, O111, O121, O103, O145, and O165 in Japan (10). However, STEC belonging to uncommon serogroups can also lead to fatal cases of HUS (11 – 13). STEC infection is not the sole cause of HUS; non-infection-related consequences are also known, referred to as atypical HUS (aHUS). The primary cause of aHUS is attributed to defects in the complement system, including mutations in complement genes or the presence of factor H autoantibodies (14). Complement-mediated aHUS is now treated with anti-complement drugs, such as eculizumab, which have shown good long-term outcomes. However, due to the high cost and adverse effects of eculizumab, including increasing the risk for meningococcal infection, it is clinically important to differentiate STEC-associated HUS and aHUS.

For the major serotypes of STEC, there are several options available for isolation and diagnosis. Since most of the major serotypes are resistant to tellurite, selective agar plates containing tellurite can be utilized to effectively isolate these STEC from specimens. Additionally, commercially available immunomagnetic beads targeting major serotypes (O-antigens) can be employed to efficiently capture the small amounts of STEC present in the specimen and separate them more efficiently. However, a method for isolating STEC belonging to minor serotypes has not yet been established, occasionally making it extremely challenging to isolate the causative STEC from patients suspected of having STEC infection.

In this study, we present a case of HUS caused by a minor STEC serotype, O76:H7. Routine diagnostic tests for major serotypes were unable to detect this serotype. Consequently, we employed modified diagnostic and isolation methods that combined PCR-based serotyping and customized immunomagnetic separation (IMS). By implementing these methods and conducting a serum agglutination test, we successfully diagnosed STEC-related HUS and isolated STEC O76:H7. Furthermore, comprehensive genomic analyses of STEC O76 in Japan and other countries unveiled its high virulence potential.

## RESULTS

### Isolation of STEC O76:H7 from an HUS case

**Fig 1:**
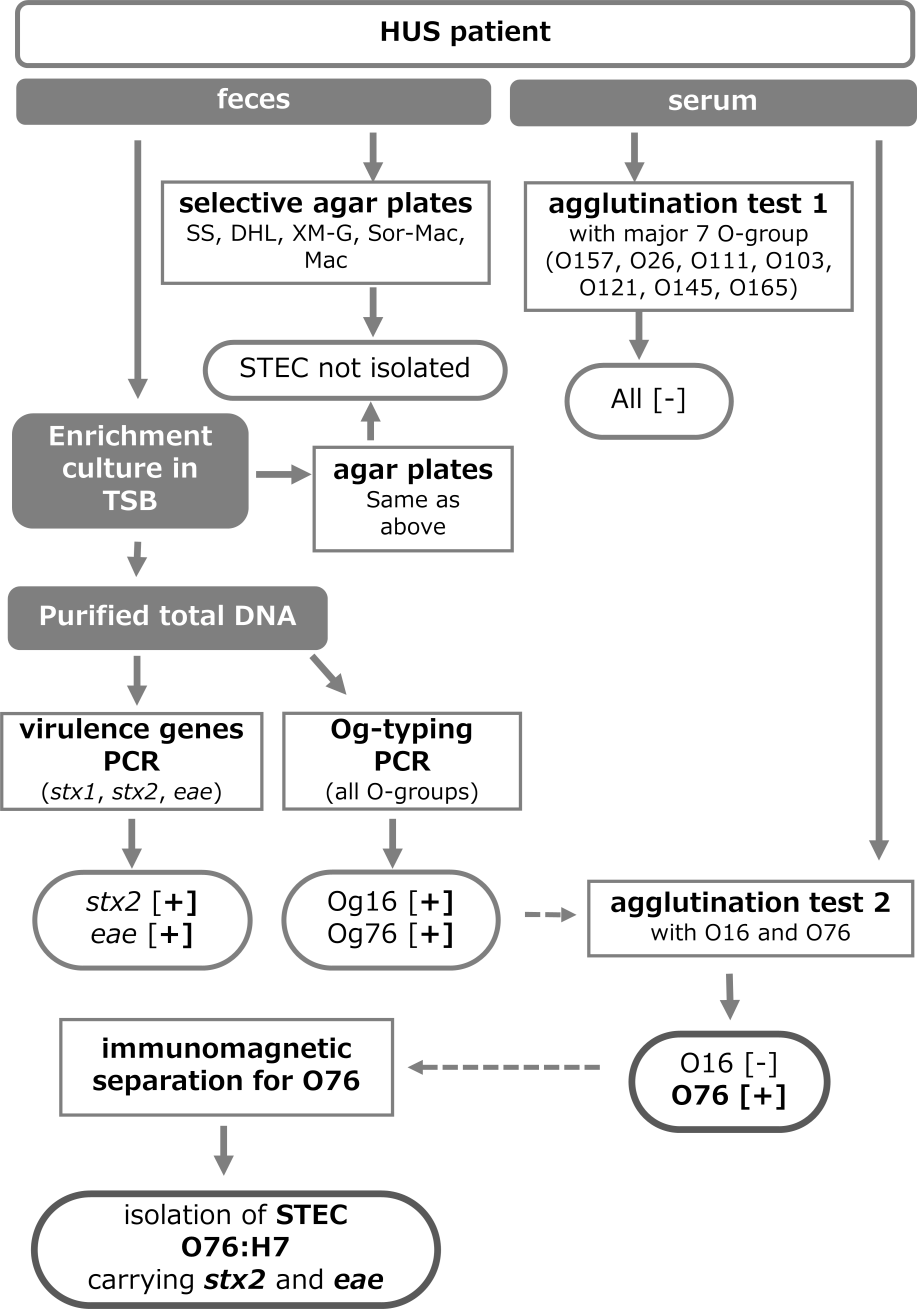
Procedures for diagnosis and isolation of causative STEC from an HUS patient.

Fecal and serum samples were collected from a 5-year-old girl who developed diarrhea followed by HUS. The workflow of detection and diagnosis is depicted in Fig. 1. First, patient serum was subjected to an assay for detecting agglutinating antibodies against *E. coli* O-antigens, including O157, O26, O111, O103, O121, O145, and O165, which consist of more than 98% of STEC isolates from HUS patients over the last 10 years in Japan (our unpublished data). The results were negative for these O-antigens. In addition, the fecal sample was cultured in a nonselectable trypticase soy broth, and then, PCR was performed to screen several virulence genes of diarrheagenic *E. coli* as well as the O-genotype of *E. coli* (13) using genomic DNA from the bacterial culture mentioned above. These PCR tests generated specific amplicons for *stx2*, *eae*, Og16, and Og76. Based upon these results, the patient’s serum was again examined to detect agglutinating antibodies against O16 and O76 antigens prepared from reference strains for *E. coli* serotyping. Agglutinating antibodies were detected only for O76. These results suggest that this case may be an STEC O76-infected HUS case. However, STEC O76 was not culturable from several agar plates as described in Materials and Methods. Therefore, magnetic beads were coupled with anti-O76 antibody, and IMS was performed to isolate STEC O76 as described in Materials and Methods. As a result, STEC O76:H7 carrying *stx2 eae* (JNE132847) was finally isolated but with extremely low efficiency.

### Whole-genome sequence analyses of STEC O76

**Fig 2:**
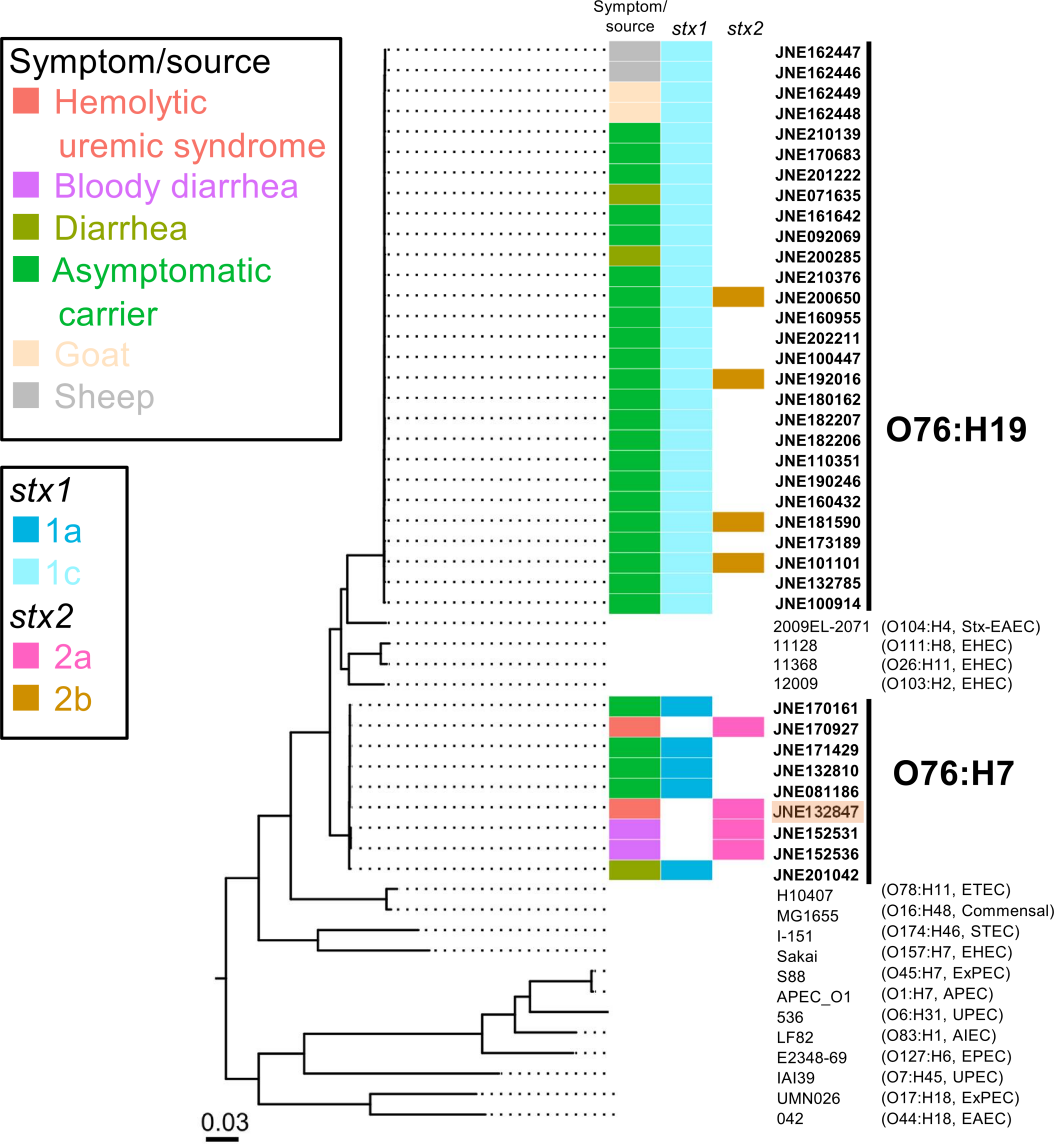
Phylogenetical relationships of Shiga toxin-producing *Escherichia coli* O76 in Japan and other *E. coli* strains. Boxes on the right represent the information on symptom of the patients and *stx* profile of the *E. coli* O76 isolates as shown in the legends. Detailed information of *E. coli* O76 isolates is shown in Table 1. STEC O76:H7 isolate from the 2013 HUS case was highlighted in orange. Serotype and pathotype information of non-O76 *E. coli* strains is shown in the parentheses and Table S1. The tree was rooted by *E. fergusonii* ATCC35469 (NCBI accession no. CU928158). Scale bar represents substitution rate per site.

To characterize HUS-derived isolates at the genomic level, whole-genome sequencing (WGS) analyses of Japanese and international STEC O76 were performed. Additional 36 STEC O76 isolates including another isolate from a HUS case and two from bloody diarrhea patients were found in the national STEC surveillance of Japan from 2007 to 2021 (Table 1). The WGS of these isolates was analyzed for phylogenetic relationships with other pathogenic and nonpathogenic *E. coli* strains (Table S1). All the STEC O76 isolates belonged to phylogenetic group B1. However, they were separated into two independent lineages, which corresponded to the H-genotype (H7 and H19; Fig. 2).

**TABLE 1:** STEC O76 isolates in Japan used in this study

Strain	Isolated year	Symptoms/source^*a*^	H genotype	Phylogenetic group	MLST	LEE	*ter*	*stx* subtype		Accession no.	
								*stx1*	*stx2*	Draft genome	Short reads
JNE071635	2007	D	B1	H19	675	–	-^*b*^	1c	–	BRVT00000000	SAMD00513697
JNE081186	2008	AC	B1	H7	795	+	–	1a	–	BRVU00000000	SAMD00513698
JNE092069	2009	AC	B1	H19	675	–	–	1c	–	BRVV00000000	SAMD00513699
JNE100447	2009	AC	B1	H19	675	–	–	1c	–	BRVW00000000	SAMD00513700
JNE100914	2009	AC	B1	H19	675	–	–	1c	–	BRVX00000000	SAMD00513701
JNE101101	2010	AC	B1	H19	675	–	–	1c	2b	BRVY00000000	SAMD00513702
JNE110351	2011	AC	B1	H19	675	–	–	1c	–	BRVZ00000000	SAMD00513703
JNE132785	2013	AC	B1	H19	675	–	–	1c	–	BRWA00000000	SAMD00513704
JNE132810	2013	AC	B1	H7	795	+	+	1a	–	BRWB00000000	SAMD00513705
JNE132847	2013	HUS	B1	H7	795	+	–	–	2a	BRWC00000000	SAMD00513706
JNE152531	2015	BD	B1	H7	795	+	–	–	2a	BRWD00000000	SAMD00513707
JNE152536	2015	BD	B1	H7	795	+	–	–	2a	BRWE00000000	SAMD00513708
JNE160432	2016	AC	B1	H19	675	–	–	1c	–	BRWF00000000	SAMD00513709
JNE160955	2016	AC	B1	H19	675	–	–	1c	–	BRWG00000000	SAMD00513710
JNE161642	2016	AC	B1	H19	675	–	–	1c	–	BRWH00000000	SAMD00513711
JNE162446	2016	Sheep	B1	H19	675	–	–	1c	–	BRWI00000000	SAMD00513712
JNE162447	2016	Sheep	B1	H19	675	–	–	1c	–	BRWJ00000000	SAMD00513713
JNE162448	2016	Goat	B1	H19	675	–	–	1c	–	BRWK00000000	SAMD00513714
JNE162449	2016	Goat	B1	H19	675	–	–	1c	–	BRWL00000000	SAMD00513715
JNE170161	2016	AC	B1	H7	795	+	+	1a	–	BRWM00000000	SAMD00513716
JNE170683	2016	AC	B1	H19	675	–	+	1c	–	BRWN00000000	SAMD00513717
JNE170927	2017	HUS	B1	H7	795	+	+	–	2a	BRWO00000000	SAMD00513718
JNE171429	2017	AC	B1	H7	795	+	+	1a	–	BRWP00000000	SAMD00513719
JNE173189	2017	AC	B1	H19	675	–	–	1c	–	BRWQ00000000	SAMD00513720
JNE180162	NA	AC	B1	H19	675	–	–	1c	–	BRWR00000000	SAMD00513721
JNE181590	2018	AC	B1	H19	675	–	–	1c	2b	BRWS00000000	SAMD00513722
JNE182206	2018	AC	B1	H19	675	–	–	1c	–	BRWT00000000	SAMD00513723
JNE182207	2018	AC	B1	H19	675	–	–	1c	–	BRWU00000000	SAMD00513724
JNE190246	2019	AC	B1	H19	675	–	–	1c	–	BRWV00000000	SAMD00513725
JNE192016	2019	AC	B1	H19	675	–	–	1c	2b	BRWW00000000	SAMD00513726
JNE200285	2020	D	B1	H19	675	–	–	1c	–	BRWX00000000	SAMD00513727
JNE200650	2020	AC	UT	H19	675	–	–	1c	2b	BRWY00000000	SAMD00513728
JNE201042	2020	D	B1	H7	795	+	–	1a	–	BRWZ00000000	SAMD00513729
JNE201222	2020	AC	B1	H19	675	–	–	1c	–	BRXA00000000	SAMD00513730
JNE202211	2020	AC	B1	H19	675	–	–	1c	–	BRXB00000000	SAMD00513731
JNE210139	2021	AC	B1	H19	675	–	–	1c	–	BRXC00000000	SAMD00513732
JNE210376	2020	AC	B1	H19	675	–	–	1c	–	BRXD00000000	SAMD00513733

^*a*^ D, diarrhea; AC, asymptomatic carrier; HUS, hemolytic uremic syndrome; BD, bloody diarrhea; NA, not available. MLST, multilocus sequence typing; LEE, locus of enterocyte effacement.

^*b*^ –, not detected.

**Fig 3:**
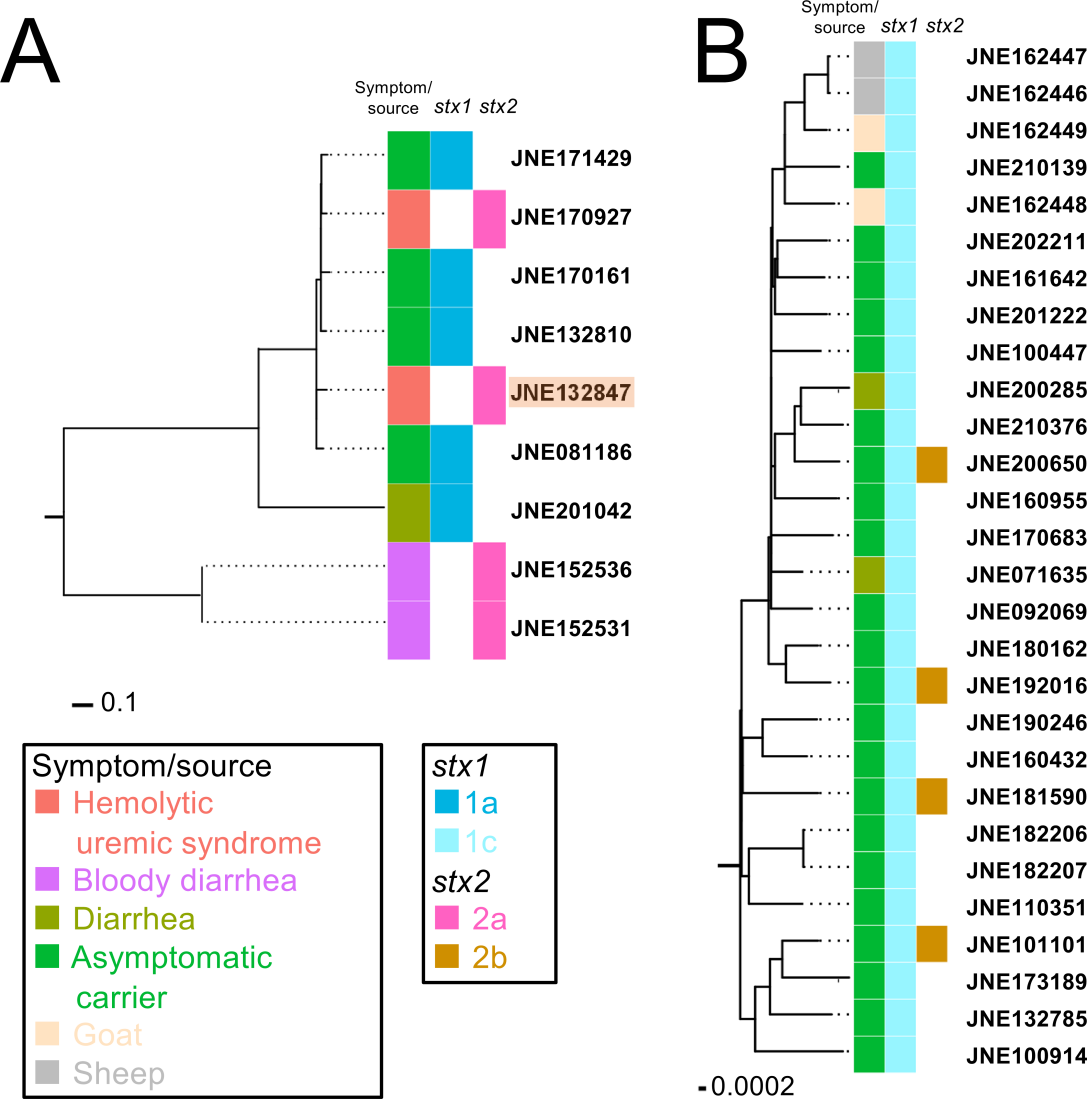
Phylogenetic relationships of Shiga toxin-producing *Escherichia coli* O76:H7 (**A**) and O76:H19 (B) in Japan. Boxes on the right represent the information on symptom of the patients and *stx* profile of the *E. coli* O76 isolates as shown in the legends. Detailed information of *E. coli* O76 isolates is shown in Table 1. STEC O76:H7 isolate from the 2013 HUS case was highlighted in orange. Scale bar represents substitution rate per site.

Four out of nine O76:H7 carried *stx2a*, and they were isolated from patients with HUS and/or bloody diarrhea (Fig. 3A). Meanwhile, all the O76:H19 isolates carried *stx1c* and most of the human isolates (22/24, 91.7%) were obtained from asymptomatic carriers (Fig. 3B). Additionally, we found that all the STEC O76:H7 isolates possessed the locus of enterocyte effacement (LEE) genes while O76:H19 did not (Table 1). O76:H19 isolates did not possess typical virulence factors for enteropathogenic *E. coli* (*bfpA*), enteroaggregative *E. coli* (*aggR*), enterotoxigenic *E. coli* (*elt* and *est*), and enteroinvasive *E. coli* (*invE*). This epidemiological and phylogenetic evidence prompted us to focus on the STEC O76:H7 lineage in further analyses.

Comprehensive phylogenetic analysis for STEC O76:H7 was performed with the Japanese isolates and WGS data on EnteroBase (http://enterobase.warwick.ac.uk/species/index/ecoli). Seventy-three entries from Asia, North America, Europe, and Africa met the requirement of high-quality WGS data as described in Materials and Methods (Table S2). All the *E. coli* O76:H7 isolates carried *eae* but did not carry *bfpA*, suggesting that the serotype is atypical enteropathogenic *E. coli*. Twenty of 82 (24.4%) of the isolates carried *stx1a* or *stx2a*; *stx* is carried in a certain lineage, consisting of isolates from Japan, Austria, the United States, and the United Kingdom (Fig. S1). The isolates of this lineage were isolated in 2013 and are constantly obtained from humans around the world. However, the *stx* profile is not concordant with their phylogeny.

### Complete genome sequence of HUS-derived STEC O76:H7

**Fig 4:**
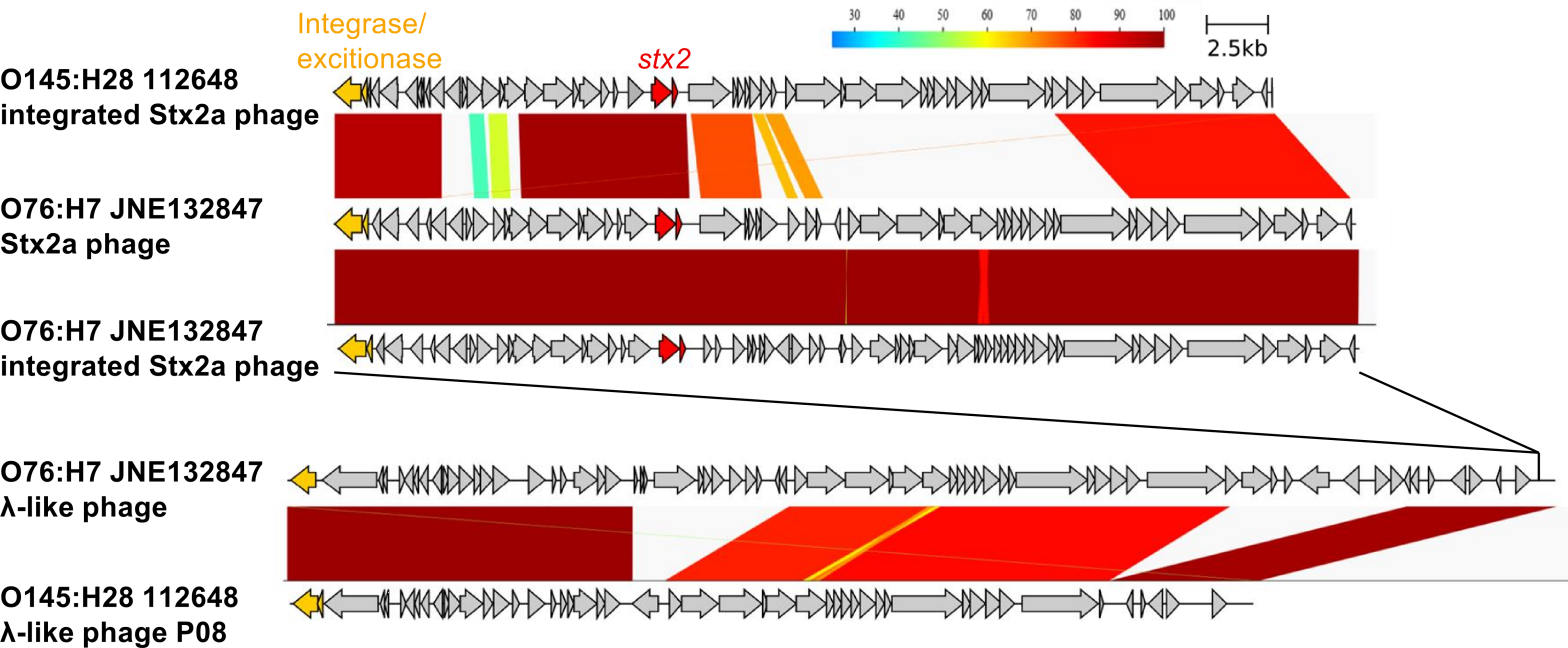
Comparison of Stx2a phages and Stx2a phage-harboring phages of O76:H7 JNE132847 and O145:H28 112648 showed difference in gene component. CDSs are shown as arrows. Similarity between the sequences was calculated by BLASTN program. Integration site of Stx2a phage of JNE132847 in the λ-like phage was indicated. This figure was generated by using GenomeMatcher v.3.06 and clinker v.0.0.25.

The complete genome sequence of JNE132847 was determined using short- and long-read sequencers. Statistics of the sequence are shown in Table 2. The genome possesses 13 prophage regions detected by PHASTER (https://phaster.ca/). Two of them were identical Stx2a phages with a similarity of 99.9%. One of them was inserted at *yecE*, and the other was inserted at the late region (downstream of *nleG*) of another phage, which is inserted at *ompW* (Fig. 4). This “prophage integrating into prophage” structure of duplicated Stx2a phages is the same as those found in the STEC O145:H28 isolates reported by Nakamura et al. (15). However, the genome structures of the phages and *attB* sequences are substantially different (Fig. 4).

**TABLE 2:** Statistics of the complete genome sequence

Statistics	Chromosome	pJNE132847_1	pJNE132847_2
Size (bp)	5,188,281	132,775	75,901
No. CDS	4,950	122	89
No. rRNA	22	0	0
No. tRNA	106	0	0
GC%	50.8	45.9	51.9
No. of CRISPRS	2	0	0
Chromosomal sequence type or plasmid Inc. type	ST795	IncFIB, IncFII	ND^*a*^
Antimicrobial resistance gene(s)	ND	ND	*bla*_TEM-1B_, *mph(A*)
Prophage region	11	2	0
Accession No.	AP028094	AP028095	AP028096

^*a*^ ND, not detected.

Using this complete genome, we expanded the genomic analyses of *E. coli* O76:H7. First, to generate the highest resolution of core genome single nucleotide polymorphism (cgSNP) analysis, cgSNPs of Japanese O76:H7 were extracted from two types of data sets and two reference genomes. As expected, the highest number of cgSNPs was obtained when only O76:H7 isolates were used for the data set and JNE132847 was used as a reference (Table S3). Interestingly, the number of cgSNPs was smallest when O76:H7 was analyzed with various serotypes despite JNE132847 being used as a reference. There were a greater number of SNPs when only O76:H7 isolates were analyzed with O157:H7 Sakai as a reference. The fewest number of cgSNPs in the diverse *E. coli* data set and an O76:H7 reference were responsible for the smallest core genome size due to the removal of recombinogenic regions.

**Fig 5:**
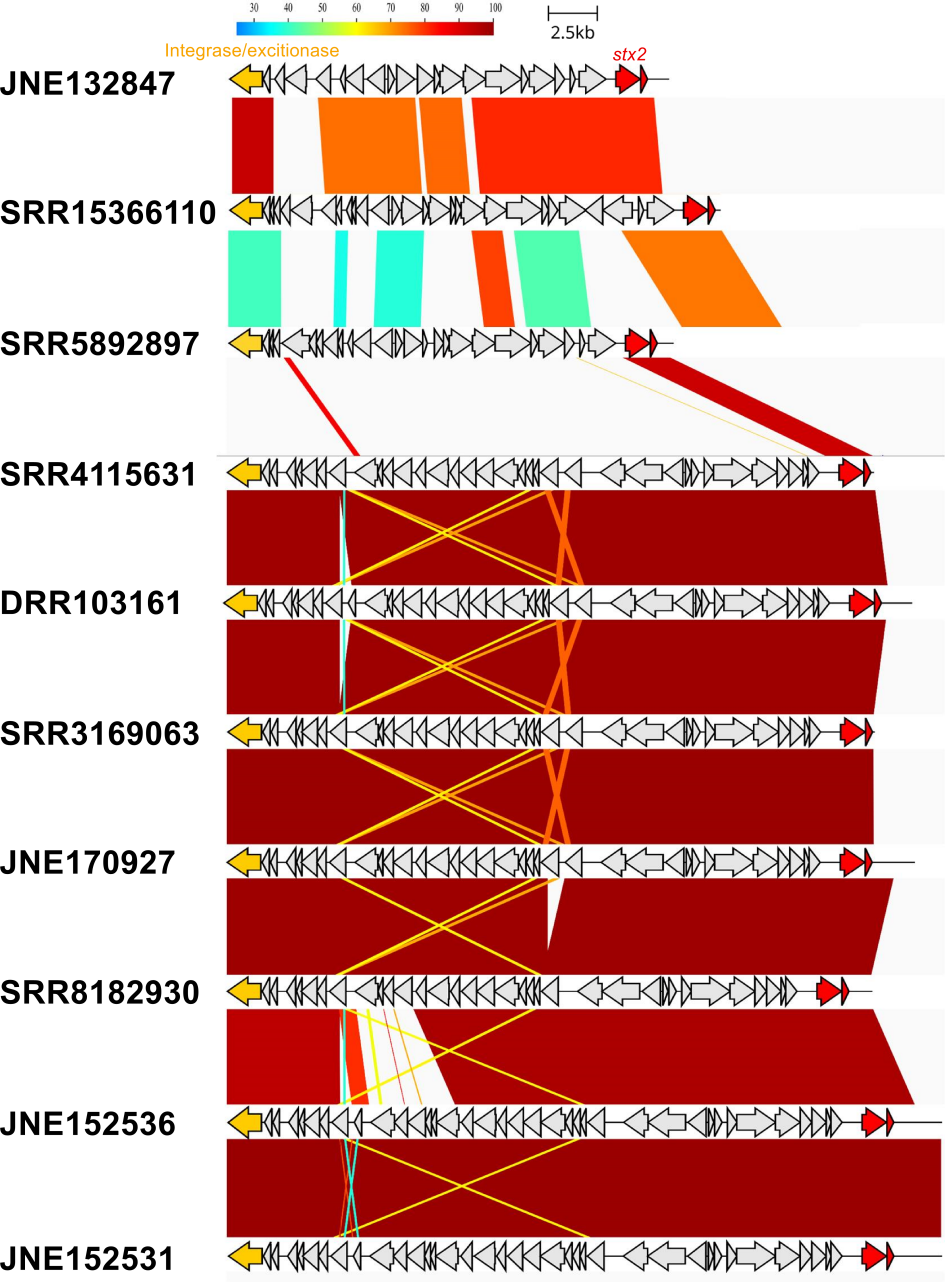
Comparison of early region of Stx2a phage of Shiga toxin-producing *Escherichia coli* O76:H7 showed the diversity of the phage. CDSs are shown as arrows. Similarity between the sequences was calculated by BLASTN program. This figure was generated by using GenomeMatcher v.3.06 and clinker v.0.0.25.

Second, the genomic structure of O76:H7 was explored in detail. The major virulence factors of STEC are *stx* and the type III secretion system encoded in the LEE region. JNE132847 carried two copies of Stx2a phages as described above. Early regions of Stx2a phage could be extracted from *stx2a*-positive O76:H7, except SRR8547517, and were compared with each other (Fig. 5). The Stx2a phage of SRR5892897 was similar to that of JNE132847 and is inserted into a prophage-like region downstream of *nleG*. The copy number of the phage and the late region remains unclear because of the fragmented draft genome. The other Stx2a phages, except SRR15366110, are highly similar and inserted into *wrbA*. The nucleotide sequence of these phages showed low similarities to that of JNE132847. The coverage of the short reads and absence of fragmentation in the upstream region of the phage suggested that only one copy of these phages is lysogenized in the genome. The Stx2a phage of SRR15366110 was inserted into the intergenic region of *mlrA* and *yehU* and showed low similarities to the other Stx2a phages. According to quantitative measurement of Stx2a production of STEC O76:H7 in Japan, the amount of the toxin produced by JNE132847 was comparable with that produced by the other isolates, which are indicated to possess only one copy of the Stx2a phage (Fig. S2). A pathogenic island encoding the type III secretion system, LEE, is highly similar to that of STEC O111:H8 (Fig. S3). According to a BLAST search of the draft genomes of STEC O76:H7, more than 90% of LEE-encoding genes are present in each isolate. Therefore, it is likely that the LEE region is conserved in this serotype. Major STEC serotypes, such as O157:H7, possess a tellurite resistance gene cluster (*terZABCDE*) on a phage-like element. Tellurite resistance is important because potassium tellurite is a selective agent in commonly used agar plates, such as CT-SMAC. JNE132847 does not possess the *ter* gene cluster. However, 4 (44.4%) and 18 (24.7%) STEC O76:H7 isolates from Japan and EnteroBase, respectively, possessed the gene cluster (Table 1). Interestingly, one STEC O76:H19 isolate from Japan possessed the *ter* gene cluster, suggesting that the gene cluster may be acquired or lost frequently.

## DISCUSSION

In this study, we have reported the first case of HUS caused by STEC O76:H7. There have been a few reports on STEC O76:H19. Meanwhile, there are scarce reports on STEC O76:H7. In Japan, no severe case by these serotypes had not been reported until 2012. However, severe cases by O76:H7 were reported in 2013, 2015, and 2017. This emerging STEC serotype requires more attention in public health. Our diagnostic procedure with IMS using O76 antibody-conjugated immunomagnetic beads and subsequent WGS analyses of STEC isolates successfully clarified the modified isolation procedure and characterized the causative isolate of HUS, respectively.

More than half of the O76 isolates in Japan belonged to H19. To date, there have been no severe cases related to this serotype in Japan, although severe cases have been reported in Spain (16) and Germany (17), and one diarrheal case was reported in Bangladesh (18). Additionally, this serotype has been isolated from sheep and goats in Spain (16), Brazil (19), and Bangladesh (20). Four isolates of STEC O76:H19 in Japan were isolated from sheep and goats. These findings suggest the possibility that sheep and goats are natural hosts of this serotype. It is also common in these studies that the majority of STEC O76:H19 possessed *stx1c* and rarely *stx2b*. While STECs with *stx2a* or *stx2d*_activatable_ have been shown to be related to severe symptoms in the infection (21, 22), there is little information on the pathogenicity of these *stx* subtypes. Additionally, this serotype lacks a major adherence factor, a LEE-encoding type III secretion system. However, all the O76:H19 isolates in Japan, but one isolate (JNE181590), possessed subtilase toxin genes (*subAB*), which are often found in LEE-negative STEC (23). This toxin or unknown virulence factors might contribute to the severe symptoms.

Severe cases in Japan were related only to STEC O76:H7, which is phylogenetically distinct from O76:H19. Therefore, we focused on this serotype for further analyses. The profile of *stx* and symptoms of the patients corresponded; severe cases were related to *stx2a*, while diarrhea patients and asymptomatic carriers were related to *stx1a*. Early literature contained few reports for this serotype except one report, in which O76:H7 isolates without *stx* were obtained from diarrheal patients in Thailand (24). A large number of genome entries for O76:H7 were found in EnteroBase. For global comparison of this serotype, WGS analyses were performed using these genomes. All the isolates possessed the LEE region and did not possess the *bfp* operon, suggesting that this serotype is atypical enteropathogenic *E. coli*. The isolates possessing *stx* belonged to a certain lineage. According to the source information, the isolates in this lineage are distributed at least in North America, Europe, and Japan. As no information about the symptoms was registered, the hazard of this serotype remains unclear. However, because some of the isolates cannot grow in certain selective media, as described below, this serotype can be underestimated. It may be worth considering this serotype in a severe STEC infection, in which the major serotypes were not detected.

For this HUS case, the initial agglutination test for major serotypes and isolation gave negative results. Although PCR results for *stx* indicated STEC infection, further examinations (i.e., serodiagnosis and isolation) were required to increase accuracy and to characterize the causative agent. Og-typing narrowed down the serotype of the causative agent, and subsequent IMS for O76 successfully enriched this serotype. Susceptibility against potassium tellurite of this serotype also made isolation more difficult. Almost half of O76:H7 lacks the *ter* gene cluster, suggesting that selective media containing potassium tellurite would have a negative effect on isolation. For this serotype, it is better to use media without potassium tellurite, in addition to selective media.

The complete genome of JNE132847 from the HUS case provided important implications for this serotype. First, a greater number of informative SNPs were obtained when the O76:H7 genome was used as a reference for cgSNP analysis than when the distantly related strain was used as a reference. When the draft genome of JNE13284 was used as a reference, the results were comparable to those using the complete genome (data not shown). A complete genome is desirable because precise SNP annotation and recombinogenic region detection can be performed. However, if it is not available, the draft genome would generate comparable results. Kwong et al. (25) reported a similar trend in WGS analyses of *Listeria monocytogenes*, which suggested that the reference genome profoundly affects the phylogeny and resolution of cgSNP analyses. In contrast, a large data set in SNP extraction leads to a decrease in informative SNPs due to a small core genome size, even if the same serotype was used as the reference. These results indicate that the data set is much more important than the reference genome. A subset of closely related strains would generate a larger core genome size and subsequently lead to a larger number of informative SNPs. Second, the complete genome of JNE132847 revealed genomic characteristics in detail. This isolate possessed two copies of the Stx2a phage. A similar “prophage integrating into prophage” structure was reported for STEC O145:H28 (15). The integration site and early region of the Stx2a phage of JNE132847 showed high similarities to those of STEC O145:H28, while the late region showed low similarities. This suggests that these “prophage integrating into prophage” structures can be abundant in the STEC population. The implications for the duplication of Stx2a phage remain unclear. The amount of Stx2a produced by the isolates with two copies of *stx2a* was not high compared with that by the isolates with one copy of *stx2a*. This result is concordant with the report by Nakamura et al. (15). Multiple copies of the Stx2a phage might contribute to maintaining the phage in the STEC population.

One of the limitations of this study is the lack of clinical information of STEC O76 isolates from the database. Our phylogenetic analysis suggested that highly virulent STEC O76:H7 exists outside Japan. Accumulation of genomic data coupled with clinical information would verify this hypothesis. The other is that the pathogenicity of the serotype was not examined *in vitro* and *in vivo*. Further experiments using cultured epithelial cell lines or experimental animals are required to this end.

In conclusion, attention should be given from a public health perspective to *stx2a*-carrying STEC O76:H7, which contributes to several severe infections. For characterization and molecular typing, obtaining the isolate still bears great importance. Conventional selective media and commercially available immunomagnetic beads cannot help isolating this serotype. Additional tests, including Og-typing, O76-specific IMS, and WGS would be helpful for effective detection and isolation of STEC O76:H7.

## MATERIALS AND METHODS

### Sample information

In 2013, a 5-year-old girl was hospitalized for vomiting, diarrhea, and stomachache. At day 4 from the disease onset, schistocytes, hemolysis, low platelet count (2.7 × 10^4^ /µL), and renal failure (BUN, 44.3 mg/dL; creatinine, 3.76 mg/dL) were observed and the condition was diagnosed as HUS. No bloody diarrhea was observed, and pathogenic *E. coli* was not detected in the hospital. Fecal and serum samples were collected from a 5-year-old girl who developed diarrhea and HUS. Serum samples were taken at days 6 and 11 from disease onset, and fecal samples were collected at day 5 and used for STEC isolation. Serum was heat inactivated at 56°C for 30 min before the agglutination test. Fecal samples were cultured in trypticase soy broth (TSB) (Becton, Dickinson and Company, Franklin Lakes, New Jersey, USA) for enrichment because selective agents can inhibit the growth of STEC (26, 27). One loopful of the culture was plated on the following agar plates: SS (Nissui Pharmaceutical Co. Ltd., Tokyo, Japan), DHL (Nissui), XM-G (Nissui), Difco MacConkey Agar (BD), and BBL MacConkey II Agar with sorbitol (BD). The genomic DNA used as a template for PCR was extracted by a DNeasy Blood & Tissue Kit (QIAGEN, Venlo, Netherlands) following the manufacturer’s instructions.

### *E. coli* Og-typing PCR

Og-typing PCR to identify the O-genotype of isolated *E. coli* or mixed culture was performed using purified DNA as described previously (28, 29). Briefly, multiplex PCR for MP-1 to MP-25 primer sets was performed followed by uniplex PCR to confirm the results.

### Serum agglutination test for STEC infection

A test to detect agglutinating antibodies against *E. coli* O-antigens in the patient’s antiserum was performed as described previously (30). Briefly, antigens were prepared from STEC O157:H7, O26:H1601, O111:H8, O121:H19, O103:H2, O165:H25, and O145:H28 isolates. The bacterial strains were grown on trypticase soy agar (Becton, Dickinson and Company) at 37°C for 18 h. The cells were suspended in saline solution and autoclaved at 121°C for 1 h. The cells were then centrifuged at 2,000 × *g* for 15 min, the supernatants were discarded, and the cell pellets were resuspended in 5 mL of saline. After washing twice with saline, the cells were centrifuged at 200 × *g* for 5 min, and the supernatants were adjusted with saline to a 3 to 4 McFarland standard by using a turbidity meter (Densimat; Sysmex-bioMérieux, Marcy l’Etoile, France). The suspensions were subsequently used as *E. coli* O antigens. The test sera were diluted 1:10 using sterile saline, inactivated by heating at 56°C for 30 min, and then centrifuged at 10,000 × *g* for 1 min. Next, 25-µL aliquots of the test sera were diluted twofold by mixing with 25 µL of sterile saline in 96-well V-shaped microtiter trays (BM Equipment, Tokyo, Japan). The highest dilution giving a clear agglutination pattern was considered the endpoint. The antibody titers were recorded as the reciprocal of the endpoint dilution of the test sera, yielding final serum dilutions ranging from 1:20 to 1:2,560.

### Preparation of magnetic beads and specific isolation of STEC O76

Dynabeads M-280 (Thermo Fisher Scientific, Massachusetts, USA) were coupled with *E. coli* O76-specific antisera (Statens Serum Institut, Copenhagen, Denmark) to prepare specific magnetic beads for the isolation of STEC O76 by the method following the manufacturer’s instructions. Briefly, M-280 beads and the O76 antibodies were incubated for 30 min at room temperature with gentle rotation. The antibody-coupled beads were washed with PBS containing 0.1% BSA three times and resuspended with PBS. Immunomagnetic separation was performed using this antibody-coupled beads and the enrichment culture.

### Isolates of STEC O76 in Japan and genome data from a public database

Additional 14 STEC O76 isolates were used for WGS analyses. These isolates were obtained from 2007 to 2021 in the national STEC surveillance in Japan. Detailed information is shown in Table 1. For global comparison, all the genome data of STEC O76:H7 in EnteroBase (http://enterobase.warwick.ac.uk/species/index/ecoli) were obtained (downloaded on 2021-Dec-6). The data matching the following criteria were used for further analyses: minimum coverage of ×40, information on collection year and place, and the “contamination” value in CheckM version 1.1.3 (31) is below 1%.

### WGS phylogeny and *in silico* typing of draft genomes

Genomic DNA was extracted with the DNeasy Blood & Tissue Kit (QIAGEN) and Genomic-tip 100/G (QIAGEN) for short-read and long-read sequencing, respectively. For short-read sequencing, genomic DNA libraries were prepared using a Nextera XT DNA Sample Prep Kit (Illumina, San Diego, CA, USA) or QIAseq FX DNA Library Kit (QIAGEN). The pooled libraries were subjected to multiplexed paired-end sequencing (300-mer × 2) using MiSeq (Illumina). The short reads were assembled using SPAdes v.3.13.0 with the “--careful” option (32). Contigs of each isolate were comprehensively characterized using an in-house BLAST-based pipeline as described previously (5). Core genome SNP-based phylogenetic relationships of STEC O76 isolates with other pathogenic and nonpathogenic *E. coli* (Table S1) were inferred by an in-house pipeline (11, 13) using BactSNP v.1.1.0 (33) with the genome of STEC O157 strain Sakai (GenBank accession No.: BA000007.3) as a reference. Repetitive regions longer than 50 bp were identified by MUMmer v.4.0.0 (nucmer, repeat-match, and exact-tandems functions) (34) and removed for further analyses, as were prophage regions. The recombinogenic regions were detected by Gubbins version 2.4.1 (35) and removed. The resultant concatenated SNP sequences were used for further analyses. Model selection and construction for a phylogenetic tree by the maximum likelihood method were performed using ModelTest-NG (36) and RAxML-NG ver. 0.9.0 with 1,000 bootstrap replicates (37). The phylogenetic tree was visualized by R version 4.1.0 with the ggtree package (38).

For long-read sequencing, gDNA was sheared by using g-TUBE (Covaris Inc., Massachusetts, USA). After blunting of the fragmented gDNA, a sequence library was prepared using the SMRTBell Template Prep Kit 1.0 (Pacific BioScience, California, USA). Size selection was performed by 20 kb template preparation using the BluePippin Size-Selection System (Sage Science, Massachusetts, USA) with a cutoff value of 15 kb. The libraries were sequenced on a PacBio RSII sequencer using C3 chemistry. Sequence reads were filtered and assembled *de novo* utilizing the PacBio Hierarchical Genome Assembly Process version 3 (39) and error corrected by pilon version 1.24 (40) with the short reads. Annotation was performed by DFAST (41) and manually curated. Genome structure was visualized by using GenomeMatcher (42) and clinker (43).

## Data Availability

We deposited draft genome sequences and short-read sequencing data into the DDBJ/National Center for Biotechnology Information/European Nucleotide Archive database (BioProject accession no. PRJDB7389; Sequence Read Archive accession no. DRA014544). Accession numbers for the complete genome of the HUS-derived isolate JNE132847 are shown in Table 2.

## SUPPLEMENTAL MATERIAL

Fig. S1, S3, S3 spectrum.02355-23-s0001.pdf Supplemental figures.

Tables S1, S2, S3 spectrum.02355-23-s0002.xlsx Supplemental tables.

ASM does not own the copyrights to Supplemental Material that may be linked to, or accessed through, an article. The authors have granted ASM a non-exclusive, world-wide license to publish the Supplemental Material files. Please contact the corresponding author directly for reuse.
