# Supplementary material for: Combined usage of serodiagnosis and O antigen typing to isolate Shiga toxin-producing Escherichia coli O76:H7 from a hemolytic uremic syndrome case and genomic insights from the isolate
Source: Microbiol Spectr. 2023 Dec 4;12(1):e02355-23. doi: 10.1128/spectrum.02355-23 (PMC10790564; doi:10.1128/spectrum.02355-23)
Supplement: Fig. S1, S3, S3 — Supplemental figures. [file spectrum.02355-23-s0001.pdf]

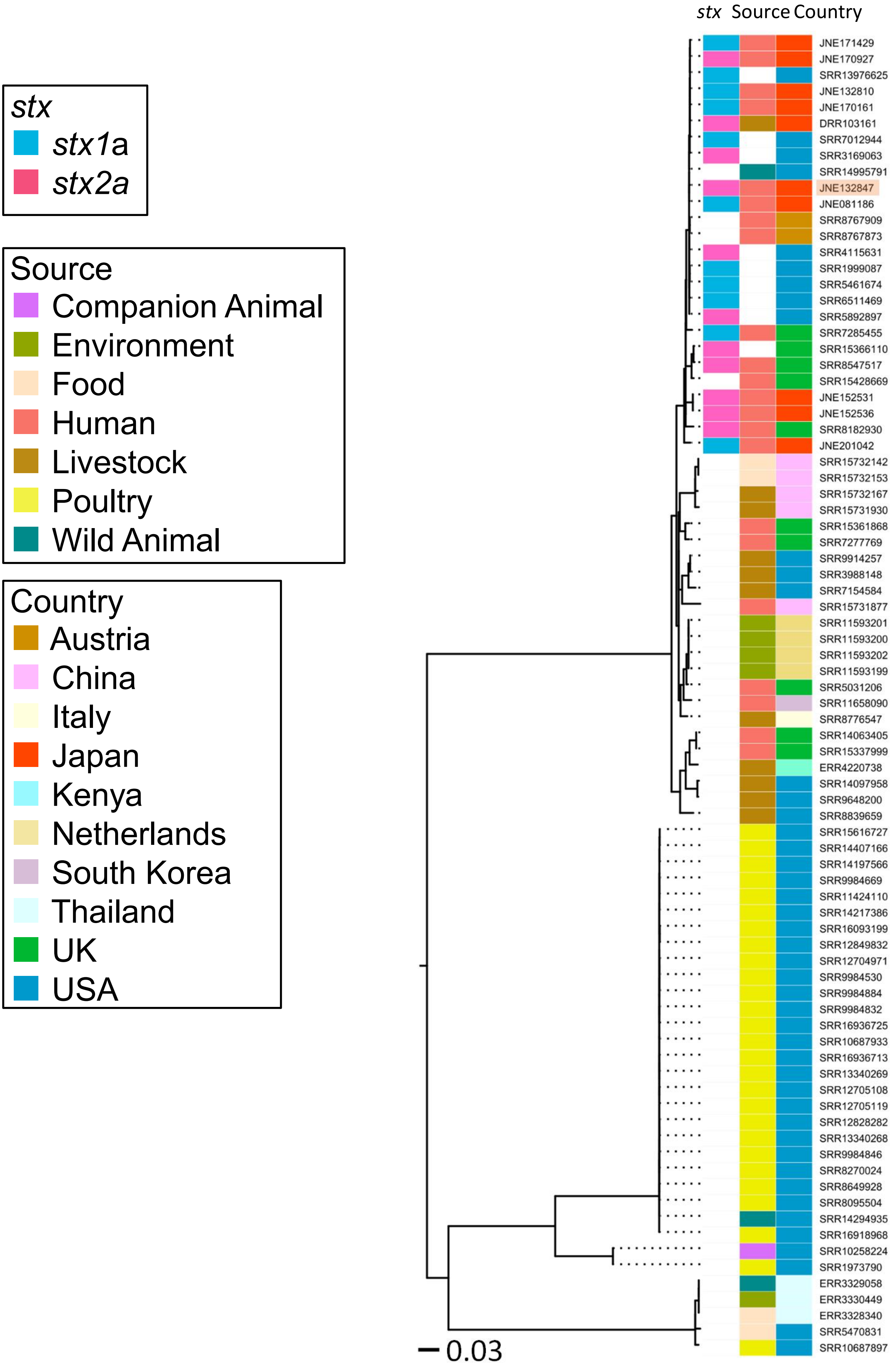

Fig S1. Phylogenetic relationships of *Eshcerichia coli* O76:H7 in Japan and in EnteroBase. Boxes on the right represent stx profile, source, and isolated country of the isolates as shown in the legends. STEC O76:H7 isolate from the 2013 HUS case was highlighted in orange. Detailed information of *E. coli* O76 isolates from Japan and EnteroBase is shown in Table 1 and Table S2, respectively. The tree was rooted by *E. coli* O157:H7 Sakai. Scale bar represents substitution rate per site.

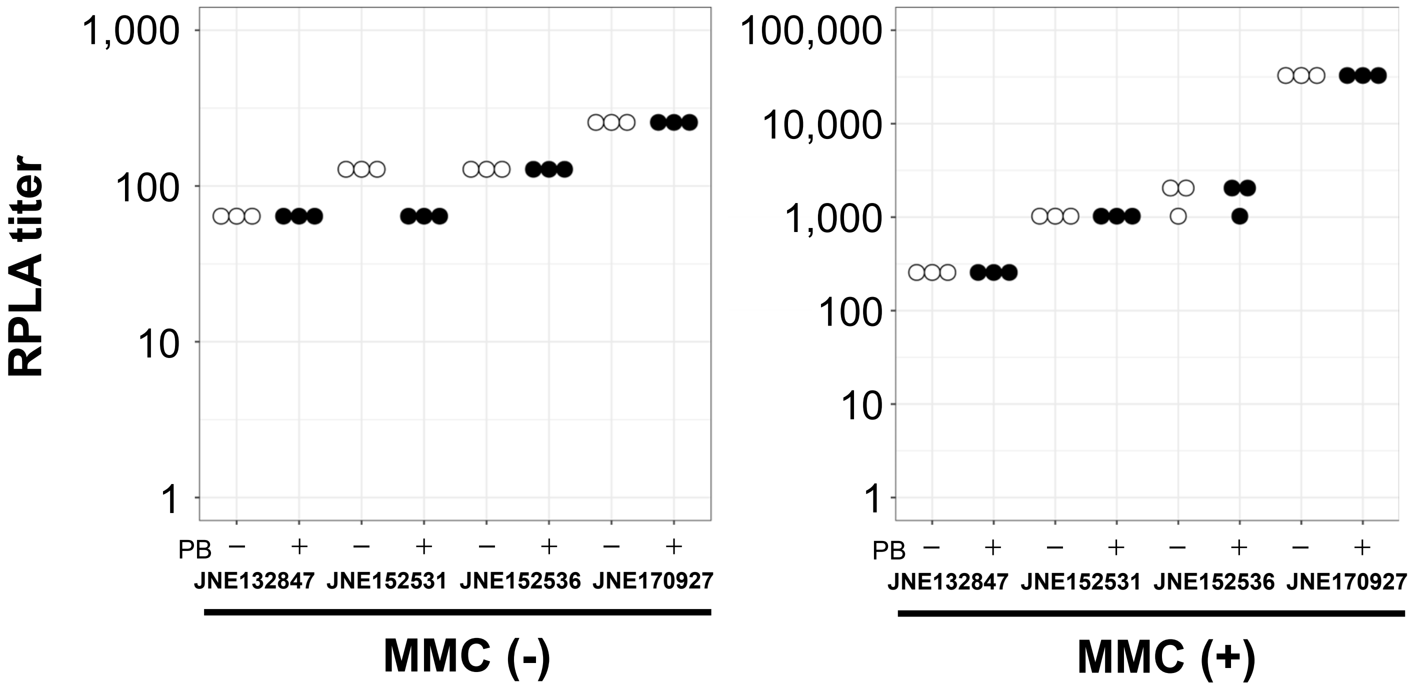

Fig S2. Stx2a titer of STEC O76:H7 measured by using reverse passive latex agglutination (RPLA) test.

RPLA titer was measured by using VTEC-RPLA (Denka Company Ltd., Tokyo, Japan) as a manufacturer's instruction. Overnight culture of STEC isolates was inoculated into fresh LB broth and incubated with shaking. When OD_{600} reached 1.0 (approximately 10^8CFU/ml), cultures were incubated with or without 1.0 μg/ml of mitomycin C (MMC). After overnight culture, bacterial cells were incubated with or without 0.5 mg/ml polymyxin B (PB) at 37° C for 30 min. The culture was centrifuged at 900 ×g 15 min and the supernatants were used for the toxin measurement. White and black circles represent triplicate results from samples without or with PB.

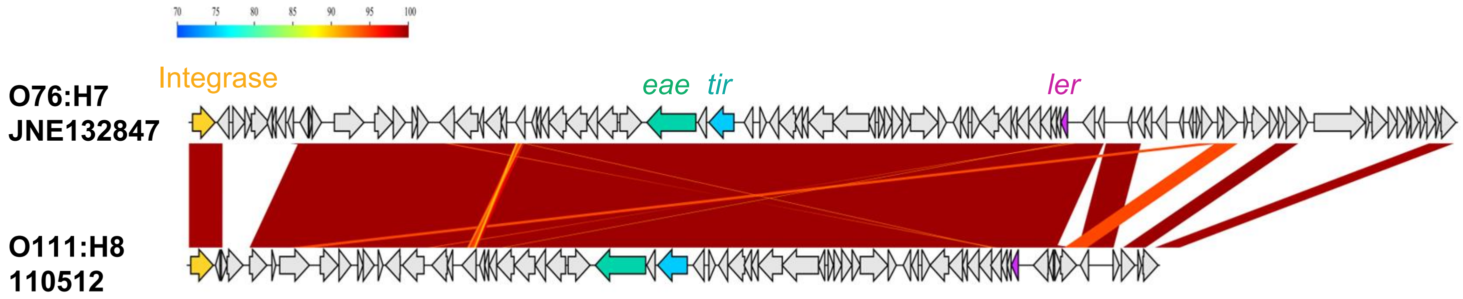

Fig S3. Comparison of locus of enterocyte effacement region of STEC O76:H7 JNE132847 and O111:H8 110512 showed high similarity of the region.
CDSs are shown as arrows. Similarity between the sequences was calculated by BLASTN program. This figure was generated by using GenomeMatcher v.3.06 and clinker v.0.0.25.
